# Supplementary figures and images for: Repurposing diphenylbutylpiperidine-class antipsychotic drugs for host-directed therapy of Mycobacterium tuberculosis and Salmonella enterica infections
Source: Sci Rep. 2021 Oct 4;11:19634. doi: 10.1038/s41598-021-98980-z (PMC8490354; doi:10.1038/s41598-021-98980-z)

Mφ2

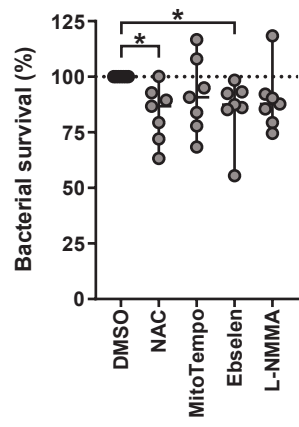

Supplementary Figure 1

Supplement: Supplementary file 1 — Supplementary Information 1. [file 41598_2021_98980_MOESM1_ESM.pdf]
