# Supplementary material for: Repurposing diphenylbutylpiperidine-class antipsychotic drugs for host-directed therapy of Mycobacterium tuberculosis and Salmonella enterica infections
Source: Sci Rep. 2021 Oct 4;11:19634. doi: 10.1038/s41598-021-98980-z (PMC8490354; doi:10.1038/s41598-021-98980-z)
Supplement: Supplementary file 4 — Supplementary Information 4. [file 41598_2021_98980_MOESM4_ESM.docx]

**Supplementary Figure Legends**

**Supplementary Figure 1 – Effect of ROS/RNS inhibitors on *Mtb* outgrowth**

CFU assay of *Mtb*-infected Mφ2 and treated with antioxidants (5 mM of NAC, 10 µM of MitoTempo, 25 µM of Ebselen or 1 mM of L-NMMA) or DMSO at equal v/v for 24 hours. Each dot represents a single donor (7 donors in total, as in Figure 5D) and depicts the mean of 3 to 6 replicates. Dotted lines indicate DMSO set at 100% with median indicated by gray bars. Statistical significance was tested using Wilcoxon matched-pairs signed rank test with post-hoc Benjamini-Hochberg correction (* = q-value <0.1).
